# From Microscopes to Monitors: Unique Opportunities and Challenges in Digital Pathology Implementation in Remote Canadian Regions

**DOI:** 10.3390/diagnostics15161983

**Published:** 2025-08-08

**Authors:** Miquela Daniel, Klaudia Nowak, Rajkumar Vajpeyi, Blaise Clarke, Andrew Evans, Charlotte Carment-Baker, Karen Weiser, Mary Martin, Nancy Girard, Kate Fyfe, Shaza Zeidan, Christine Bruce, George M. Yousef

**Affiliations:** 1 Laboratory Medicine Program, University Health Network, 200 Elizabeth Street, Toronto, ON M5G 2C4, Canada; miqueladaniel@hotmail.com (M.D.); klaudia.nowak@uhn.ca (K.N.); rajkumar.vajpeyi@uhn.ca (R.V.); blaise.clarke@uhn.ca (B.C.); charlotte.carment-baker@uhn.ca (C.C.-B.); karen.weiser@uhn.ca (K.W.); shaza.zeidan@sickkids.ca (S.Z.); christine.bruce@uhn.ca (C.B.); 2 Department of Laboratory Medicine and Pathobiology, University of Toronto, Toronto, ON M5S 3K3, Canada; 3 Mackenzie Health, Toronto, ON L4C 4Z3, Canada; andrew.evans@mackenziehealth.ca; 4 Timmins and District Hospital, Timmins, ON P4N 0A2, Canada; mary.martin@blancheriverhealth.ca (M.M.); ngirard@tadh.com (N.G.); kfyfe@tadh.com (K.F.)

**Keywords:** artificial intelligence, whole slide imaging, digital pathology, remote diagnosis, digital pathology implementation, digital health, telepathology

## Abstract

**Background/Objectives:** Digital pathology has the potential to revolutionize pathology diagnostics, especially in geo-graphically isolated and underserved regions. By leveraging technology, telepathology, and integration with computer-aided diagnostic tools, digital pathology can improve access to prompt and accurate diagnostics. **Methods:** Our key steps to implementing digital pathology and transitioning operations to a digital network are assessing existing infrastructure, identifying gaps in connectivity and resources, and creating a workflow tailored to the needs of the healthcare system. **Results:** We present an approach of implementing digital pathology in Timmins, Northern Ontario, Canada, focusing on addressing regional disparities and the improvements that come alongside utilizing digital pathology. Our results show that digital pathology can provide prompt, efficient and better-quality diagnostic services to rural and un-deserved areas, improving patient care and outcomes. It also represents a cost-effective option with savings from eliminating travel costs, courier costs and additional operational efficiencies. **Conclusions:** Implementing digital pathology in rural settings presented with challenges related to infrastructure, technical abilities, workforce readiness, cost and other aspects involved in transitioning from traditional microscopy to a fully digital pathway. Digital pathology systems can help ensuring seamless data flow and improving overall healthcare delivery. Telepathology also allows pathologists to provide diagnostic services from a distance, which is particularly beneficial in areas with a shortage of pathologists.

## 1. Introduction

Histopathology diagnostics play an important role in patient care, especially in cancer. Delivering pathology services in remote areas, however, has multiple limitations, including a lack of resources, a shortage of sub-specialized professionals, and difficulty in recruiting qualified personnel, from technical staff to pathologists [1]. The advancement of digital pathology as a primary diagnostic tool introduced a transformative change in the clinical diagnostics field [2]. The literature suggests that providing telepathology services in underserved and rural areas leads to cost savings and a faster and better service [3]. Additionally, digital pathology is a pivotal movement towards the advancement of AI-influenced pathology [4,5]

The University Health Network (UHN) is a multi-center academic tertiary hospital with over 1200 beds located in Toronto, Ontario, Canada. Its Laboratory Medicine Program provides services to 32 partner hospitals of various capacities across Ontario. Since 2004, the UHN has been a pioneer in digital pathology, demonstrating a strong history of validation and incremental implementation projects [6,7]. The UHN began its transfer toward digital pathology in 2004 by introducing a robotic microscopy telepathology system for primary frozen section diagnoses. By 2006, the UHN transitioned to a virtual slide telepathology system.

**Figure 1:**
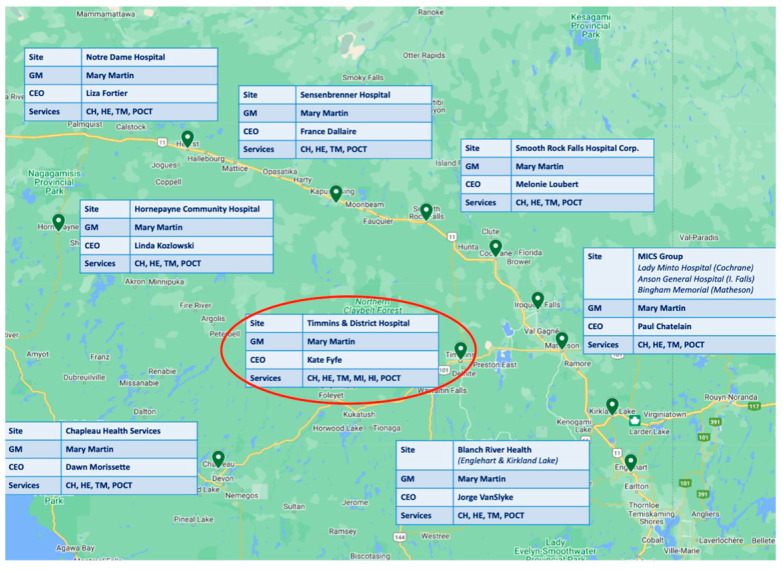
A map showing the Timmins and District Hospital (TADH) cluster and respective information per site. GM: general manager, CA: chemistry, TM: transfusion medicine, POCT: point of care testing, HE: hematology. Timmins is circled in red. Laboratory medicine services are shared among members of the cluster.

The UHN has provided exceptional leadership in laboratory medicine services including pathology, setting a high standard across Northern Ontario, including Timmins. Timmins, a surgical center located in Northern Ontario, is a vibrant community with rich history and diverse culture [8]. With a population of approximately 41,000 people, the city plays a critical role in the healthcare system of the region, serving as a hub for medical services for surrounding communities. Timmins and District Hospital (TADH) (Figure 1) is the primary healthcare facility in the area, providing a full spectrum of services, encompassing emergency care, surgical procedures, and specialized treatments [9].

In this study, we share our experience in digitizing pathology services at TADH and explore the impact of digital pathology implementation in a regional healthcare setting, focusing on TADH’s efforts to integrate advanced technologies into its diagnostic and treatment processes.

## 2. Materials and Methods

### 2.1. Assembly of Digital Pathology Team

The first step towards implementation was to establish an inter-institutional team with members from the UHN and TADH. This team consists of the project management team, leadership from both hospitals, pathologists, Medical Laboratory Technologists (MLTs), Medical Laboratory Technicians (MLAs), IT services in both institutions and representatives from the hardware (scanners) and software (image management system) vendors.

### 2.2. Resource Assessment

The initial task of the digital pathology team was to assess the resources and infrastructure available at Timmins. This included evaluating the IT infrastructure, hardware needs, and image storage capabilities. We also assessed space and the compatibility between the two different HISs at the UHN and Timmins (Meditech vs. Epic) and two laboratory information systems (Meditech vs. Beaker Anatomical Pathology).

### 2.3. Financial Assessment

An important step that was required before the implementation was to assess the finances of the project, including costs of infrastructure (scanners, image management software, IT requirements for digital pathology, and digital storage), training at TADH to ensure proficiency, and added expenses for scanning and digital QA steps. Digital pathology required active IT communication between two hospitals, translating to additional costs including improving internet and bandwidth and integrating the hospital and laboratory information systems. It was also essential to address the cost-sharing model and define the responsibilities of each partner.

### 2.4. Infrastructure Requirements

The digital pathology team assessed a few additional infrastructure requirements for setting up a remote pathology service at Timmins. Providing digital pathology services required, in addition to the IT requirements above, additional space, workflow changes, capital equipment purchase, potentially hiring new employees, and training in different aspects of digital pathology.

## 3. Results

**Figure 2:**
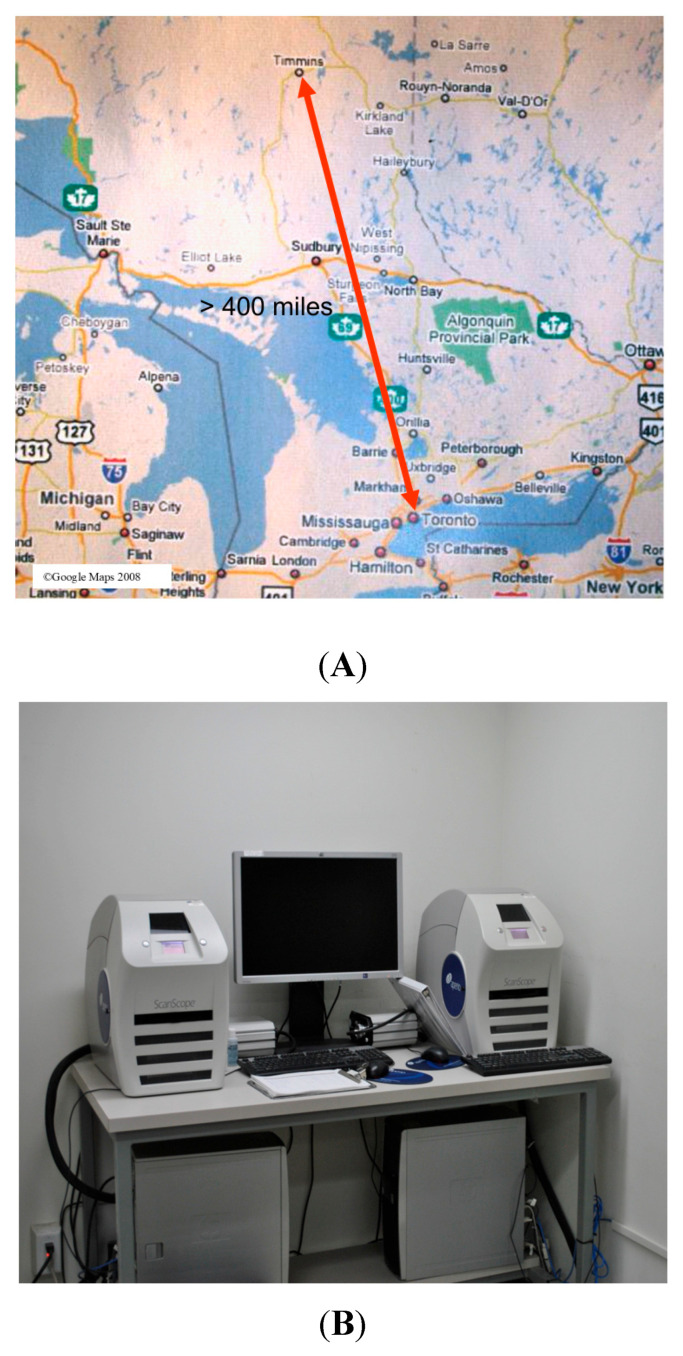
Remote intra-operative consultation for TAHD. (**A**) The distance between UHN and TADH is indicated by the arrow. (**B**) Image scanners were used to give instant access to sub-specialized pathologists at Toronto General Hospital to provide timely intra-operative consultation for Timmins.

Prior to digital pathology, UHN pathologists were required to travel to Timmins and work in rotations to provide a service to the area. After the service was established, we started our digital journey by providing digital intra-operative (frozen section) consultation (Figure 2A,B).

**Figure 3:**
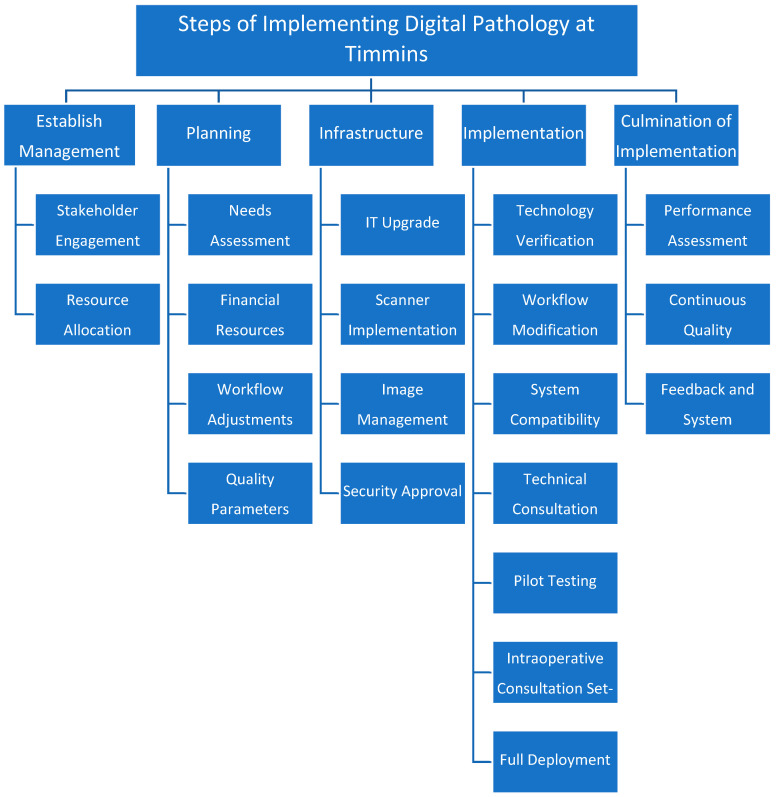
A summary of the steps of digital pathology implementation at TADH. Please note that some of these steps were overlapping.

Consultations were physically shipped to Toronto, but over time, a Leica/Aperio AT2 Turbo scanner was introduced in TADH, allowing us to handle the majority of the scanning locally at Timmins. Finally, a decision was made to move into a fully digital system that was approved by the two institutions. The stepwise process is shown in Figure 3.

We followed the same implementation protocol that we used for our main location with few modifications [9,10] to account for varying resources, infrastructure, and needs at TADH.

The first step was the establishment of the team. The project management team was formed of the Project Lead Team, the Medical Director of Timmins, the Senior Operational Directors of both the UHN and Timmins, and representatives of the IT departments of both institutions.

**Figure 4:**
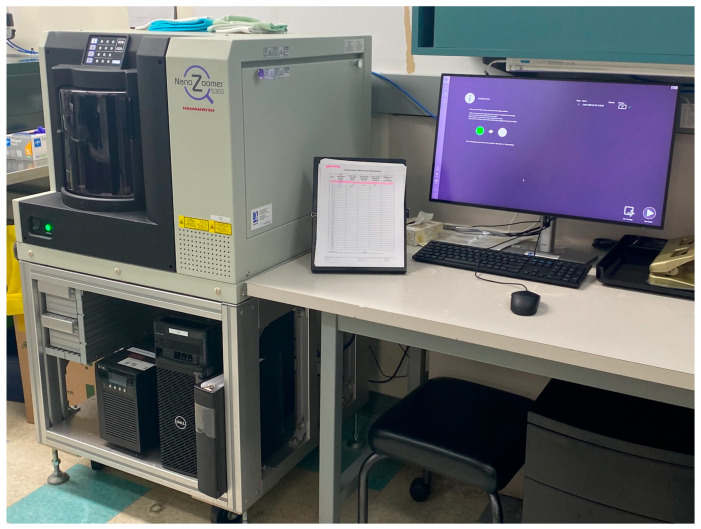
The current updated scanner in Timmins.

*Scanner and image management systems:* Considering case volumes at TADH, a new high-throughput NanoZoomer S360 scanner (Quorum Technologies, Toronto, ON, Canada) was purchased (Figure 4). The older lower capacity scanner was kept as a backup and to be used for frozen sections (intra-operative consultation). In case of scanning failure or delays, glass slides can be utilized as a second backup. The scanner works regular hour shifts. The scanner purchase cost was provided by the UHN, while Timmins covered its own ongoing operation expenses. The scanner was connected to Synapse Pathology (Fujifilm, Tokyo, Japan), which is used across all our partner sites.

*The validation process:* An important step was to perform a technical validation of the scanner at TADH to ensure that the instrument/software met the expected specifications. We followed a holistic approach for validation that ensured testing technical connectivity and compatibility between the HIS and LIS at both institutions, as detailed in our recent publication [11], and applied the same quality and performance assessment across all sites including Timmins.

Maintaining consistent quality and performance across all sites is crucial for the success of digital pathology systems. Variability in performance can undermine the reliability and credibility of the system, including regular audits and performance assessments, which are necessary to ensure consistent adherence to standards.

*Workflow modifications:* In addition to scanners and image management system connection, TADH implementation required the modification of space to accommodate the new scanner and the extra time added for lab assistants to scan all slides.

An MLT was additionally trained to gross more complex cases that were previously shipped to Toronto and to obtain images of gross specimens to be included in the digital archives. A medical laboratory assistant was also trained to process biopsies. Additional digital pathology training was needed for the rest of the MLTs, lab technicians and administrative lab personnel.

*Setting up frozen section protocol:* To maximize the value of digital pathology at TADH, we also updated our digital intra-operative consultation protocol where frozen section specimens are processed locally and immediately scanned and uploaded to the main UHN cloud. The frozen section system is now connected to the main image management and storage system at Toronto General Hospital. A special modification of keeping the frozen section slide for 16 min under a bench sweep was added to ensure specimen drying and ensure that it would not negatively affect the scanner by leaking residues.

### The Impact of Digital Pathology at Timmins

The impact of digital pathology implementation on service quality was significant. The main benefits of digital pathology are shown in Box 1. After the implementation of digital pathology, slides could be viewed at the UHN within a few hours and the average turnaround time for cases was reduced significantly. Additionally, digital pathology resulted in significant cost savings. We collectively measured different parameters to assess the financial impact including calculating infrastructure and direct/indirect savings per case. Overall, cost savings resulted from reducing/nearly eliminating the shipment of glass slides; further cost savings are captured through the reduced length of stay at TADH resulting from earlier results and treatment interventions. Added to this is the reduction in lost or broken glass slides during transportation.

Box 1 **The benefits of digital pathology implementation at TADH.**

- Enhanced accessibility and collaboration;
- Improved workflow efficiency;
- Helped in overcoming pathologists’ shortage due to them retiring or leaving the region;
- Reduced challenges related to distance, travel, weather, and logistics;
- Digital data preservation enabled easier access to past reports;
- An all-digital platform centralized data for deeper regional health analysis;
- Educational and research opportunities;
- Potential integration with AI;
- Reporting times decreased significantly;
- Greater flexibility for pathologists to report cases at their convenience.

The introduction of digital pathology was also aimed at addressing the shortage of pathologists retiring or leaving the region, as well as alleviating challenges related to distance, travel, weather, and logistics. Also, instead of relying on a generalist pathologist who would be more likely to send cases for a secondary consultation, slides are now digitally reviewed by specialized pathologists, greatly reducing the need for a second opinion and reducing extra billing.

Another advantage of digital pathology lies in the shared incremental capacity across the system. By pooling resources, all hospitals in our network benefit from a more efficient and collaborative approach. Instead of each site depending on local pathologists to handle the workload, cases can now be strategically distributed across a network of specialists ensuring patients receive expert reviewed pathology reports. Table 1 provides a comparison summarizing pathology practices during the six months before and after the implementation of digital pathology in Timmins.

## 4. Challenges

Implementing digital pathology involves several critical steps to ensure a smooth transition from traditional microscopy to a fully digital pathway. Throughout this process, several unique challenges were faced, as summarized in Box 2. One of the primary barriers was cost. Digital pathology required a significant upfront investment including infrastructure, scanners, image management software, technical support, training, quality management systems [12,13]. Ensuring compatibility and integration with existing laboratory information systems (LISs) and hospital information systems (HISs) was not only a complex task but a costly one as well [12,14,15].

Box 2 **The challenges of digital pathology implementation at TADH.**

- High initial costs;
- Ensuring the consistency of validation requirements among different sites;
- Professional hesitation;
- Data security and privacy;
- Limited expertise;
- The need to update infrastructure;
- Integration between different HISs and LISs;
- Alignment among partners for a unified approach.

Additionally, disruption to traditional workflows was a significant hurdle. The hybrid glass–digital workflow where both formats that were initially used concurrently had further hindered the adoption of going digital. During this initial period, it had created inefficiencies and made it appealing for pathologists to revert to the traditional glass slide workflows. Moreover, technological challenges such as image quality issues were hindering factors at the beginning. Overcoming these barriers required structured implementation strategies that prioritized training, quality assurance, and workflow optimization [16]. Effective communication and change management strategies were necessary to secure buy-in from all parties involved [12,14,17,18].

Bandwidth limitations and inter-institutional firewall issues were also initially impeding smooth image transmission [15,16,19]. Ensuring data security and privacy requirements were met was a significant concern, particularly when using cloud-based storage solutions because of transmission data and breach risks, as documented in recent publications [12,14,20,21]. One of the key concerns was the large file size of whole slide images (WSIs), which required high-speed network infrastructure to facilitate real-time consultations and prevent delays. The system at TADH was built with multiple connectivity options to ensure uninterrupted telepathology services, although the reliability of internet connectivity still remains a potential risk.

The complexity of establishing and developing a quality management system tailored to digital pathology operations was equally important. This included standard operating procedures, quality control measures, and continuous monitoring protocols to ensure consistent quality and patient safety [13]. Due to limited technical expertise and the need for training programs for pathologists and laboratory staff, it was necessary to ensure proficiency in using digital pathology systems [12,14,20].

## 5. Discussion

Digital healthcare has been proven to be a valuable tool for underserved areas. Virtual care alludes to the use of digital technology and telecommunications tools to provide treatment and counseling remotely. This includes services such as telemedicine, remote disease monitoring and virtual health visits, allowing health professionals to care for patients without the need for in-person visits, making care more accessible and convenient.

Virtual diagnostics comprise an important dimension in virtual care. Digital pathology has the power to revolutionize rural pathology by allowing quicker diagnoses, enhancing local expertise, and improving accuracy through computer-aided diagnostics [1].

The benefits of providing telepathology services for remote areas are vast [22]. Rural areas have significantly fewer doctors and specialists per capita compared to urban areas, highlighting the need for technological solutions to bridge the healthcare gap [23,24,25]. Without digitalization, and given the limited availability of professional pathology services in rural communities, patients often seek diagnostic reports from professional pathologists at tertiary hospitals [23]. Additionally, telepathology helps reduce waiting times and improves access to pathology services by overcoming geographical barriers [3].

Telepathology gives pathologists flexibility by allowing them to access cases from afar. It also enables rural clinics to share digitized slides instantly with specialists worldwide, enabling quicker diagnoses and access to specialized care.

The economy of digital pathology is complex and needs to be navigated carefully [26]. It should be noted that in cost assessments, other indirect cost-savings should be included in the formula including travel cost saving, travel time, and waiting time in the central hospital. These were found to be significantly lower in digital telepathology services than conventional services in Bangladesh [3].

Our results show the importance of developing customized implementation plans that address the specific requirements and constraints of each site, including selecting appropriate hardware, software, and connectivity solutions. It also highlights the need for training and support through on-site and virtual training and support to ensure that staff at peripheral sites are adequately prepared during the transition.

In our case, implementing and expanding digital pathology across Northern Ontario will additionally enhance the collection of pathological data, creating a more comprehensive database for the northern region. By transitioning to an all-digital platform, data can be centralized and accumulated from multiple locations, allowing for a deeper analysis and insights into regional health trends. Such a repository can help us to gain a clearer understanding of disease patterns that were previously hidden due to limited, fragmented data from individual referrals. This approach will allow us to make more informed decisions and tailored treatment strategies and improve public health outcomes throughout Northern Ontario.

More studies are needed to highlight the advantages and limitations of the digital pathology implantation of rural areas. We acknowledge the limitations of this study which include its single geography, lack of resized clinical outcomes for comparison, and reliance of estimated costs rather than mathematical calculations. Additionally, longer-term follow ups are needed to validate the cost savings and the impact of clinical service.

### Global Impact Pathology, a Multidisciplinary Action!

Rural and remote cancer care varies, with patients in these areas often have worse outcomes than those in urban areas. This is even greater in lower-income countries with limited healthcare resources [27,28]. Key factors include limited access to lab medicine and cancer diagnostics. In order to achieve a global impactful equitable management scheme for serious diseases, including cancer, digital pathology can play an important role. This must be a collaborative multidisciplinary effort between different institutions in developed and developing countries. Engaging participation from industrial and pharmaceutical companies’ involvement would also be essential for the proper utilization of precision medicine therapy that is expensive and needs to be focused on those who are likely to respond. Philanthropy is another key player towards global pathology outreach; the more resources and time put towards supporting these causes can benefit the healthcare system.

## 6. Conclusions and Future Directions

Rural and remote digital pathology is relatively underrepresented in the literature and future research should be designed with this in mind. Areas of focus should include a systematic review of digital pathology in rural and remote healthcare, the development of deep learning tools for specialized applications, and the use of digital pathology for training and mentorship. Moreover, research should analyze current and future infrastructure to assess its ability to support digital pathology in these settings.

## Figures and Tables

**Table 1:** The impact of digitization on workflow at Timmins cluster.

Parameter	Before Full Digital Implementation	After Full Digital Implementation	Notes
% of Cases Digitized	10%	100%	All pathology cases are now digitized, eliminating the need for transporting physical slides between institutions.
Average Turnaround Time (TAT) for Biopsy Diagnosis	4 business days	~2 business days	Whole slide images were accessible 1–4 days earlier than glass slides, improving diagnostic efficiency.
Average TAT for Resection Diagnosis	10 business days	~8 business days	This especially improved in cases where prior slides were digitized and accessible during case review.
Intra-Operative Consults (Frozen Section)	Limited by on-site pathologist availability	Continuous remote support via digital pathology	Real-time frozen section consultations became available even without an on-site pathologist.
Organ Site Specimen Digitization	GI = 50%	GI = 100% Breast = 100% GU = 100% Gyne = 100% Heme = 100% Derm: 100%	Full digitization now covers gastrointestinal (GI), breast, genitourinary (GU), gynecological (Gyne), hematopathology (Heme), and dermatopathology (Derm) specimens.
Digital Integration with Laboratory Information System (LIS)	No	Yes	This enables simultaneous access to clinical data and digital images for pathologists across locations.
Lab Employee Number	Same	Same	No additional FTEs were needed; existing roles were restructured to support digital operations.
Annual Cost Savings	CAD 0	~CAD 131,000–CAD 175,000	Estimated savings include CAD 60K in travel, CAD 45K in accommodation/meals/car rental, CAD 26K in courier costs, and additional operational efficiencies.
5-Year Projected Cost Savings	CAD 0	~CAD 1.3 million	This is based on sustained reductions in transport, storage, and personnel-related travel over a 5-year period.
Estimated Implementation Cost	N/A	~CAD 150,000	Initial costs included purchasing a demo scanner, installing the scanner, training staff, and procuring dedicated IT support.
Estimated Return on Investment (ROI)	N/A	Achieved within 1–2 years	The cost savings realized from reduced travel, accommodation, and courier expenses offset the initial investment within a short period.

## Data Availability

No new data were created or analyzed in this study. Data sharing is not applicable to this article.

## Abbreviations

TADH: Timmins and District Hospital. LMP: Laboratory Medicine Program.
